# Multi-phase separation in mitochondrial nucleoids and eukaryotic nuclei

**DOI:** 10.52601/bpr.2023.220018

**Published:** 2023-06-30

**Authors:** Qi Long, Yanshuang Zhou, Jingyi Guo, Hao Wu, Xingguo Liu

**Affiliations:** 1 CAS Key Laboratory of Regenerative Biology, Joint School of Life Sciences, The Sixth Affiliated Hospital of Guangzhou Medical University, Qingyuan People’s Hospital, Guangzhou Medical University; Guangzhou Institutes of Biomedicine and Health, Chinese Academy of Sciences, Guangzhou 510530, China; 2 Guangdong Provincial Key Laboratory of Stem Cell and Regenerative Medicine, China-New Zealand Joint Laboratory on Biomedicine and Health, CUHK-GIBH Joint Research Laboratory on Stem Cells and Regenerative Medicine, Institute for Stem Cell and Regeneration, Institute for Stem Cell and Regeneration, Guangzhou Institutes of Biomedicine and Health, Chinese Academy of Sciences, Guangzhou 510530, China; 3 Centre for Regenerative Medicine and Health, Hong Kong Institute of Science & Innovation, Chinese Academy of Sciences, Hong Kong SAR, China

## Abstract

In mammalian cells, besides nuclei, mitochondria are the only semi-autonomous organelles possessing own DNA organized in the form of nucleoids. While eukaryotic nuclear DNA compaction, chromatin compartmentalization and transcription are regulated by phase separation, our recent work proposed a model of mitochondrial nucleoid self-assembly and transcriptional regulation by multi-phase separation. Herein, we summarized the phase separation both in the nucleus and mitochondrial nucleoids, and did a comparison of the organization and activity regulating, which would provide new insight into the understanding of both architecture and genetics of nucleus and mitochondrial nucleoids.

## INTRODUCTION

The compartmentation in cells is critical for carrying multiple functions (Boeynaems* et al.* 2018). It is well known that the inner membrane system segregates space in cells into organelles, such as the endoplasmic reticulum, Golgi apparatus, lysosomes, endosomes and so on. These organelles concentrate special factors to generate small reaction centers, which provide spatiotemporal control over cellular materials, metabolic and signals for cells in carrying multiple functions and pathways. Despite the inner membrane system, phase separation could also concentrate biomolecule independence of the membrane systems, which greatly expands the compartmentation in cells and reduces the threshold for generating small reaction centers (Banani* et al.* 2016, 2017; Riback* et al.* 2020).

Phase separation serves as a biomolecular condenser, which concentrates biomolecules to form droplet-like or gel-like structures (Banani* et al.* 2017; Shin and Brangwynne 2017). It is a kind of self-assembly driven by the intrinsic property of the biomolecules, such as weak interaction beneath intrinsically disordered regions (IDR) or multivalent effect between molecule interaction (Alberti* et al.* 2019). Not only proteins, DNAs, RNAs and even small molecules, such as dNTPs and drugs could be concentrated by co-phase separation (Klein* et al.* 2020; Wang* et al.* 2018), which provides the substrates, enzymes or cofactors for the efficient reaction in the small reaction centers generated by phase separation.

Phase separation provides the driving force to concentrate biomolecules into membraneless organelles, such as stress granules, cell junctions, signal transduction, transcription complex and so on (Banani* et al.* 2016). It is also reported to take part in the condensation of chromatin, enhancers, nuclear speckles, heterochromatin and even the architecture of nuclei, such as the generation of the nucleolus (Sabari* et al.* 2020). Nuclei are the largest organelle in cells and play critical important and multiple roles in DNA storage, replication and transcription, ribosome assembling, *etc*. The compartmentation by phase separation is critical for the nuclei in carrying multiple roles.

Mitochondria are the only semi-autonomous organelle in mammalian cells, owing a circle DNA, which plays critical roles in energy production, metabolism, apoptosis, and so on. Mitochondria DNA is organized into nucleoids by TFAM, one of the most abundant proteins in mitochondria. The crista plays a critical role in the compartmentation of mitochondria for metabolism and energy production (Cogliati* et al.* 2013; Frey and Mannella 2000). However, there are rare reports about the compartmentation in mitochondrial nucleoids, which share many commons with the nuclei, as well as some unique features. Recently, we reported that phase separation drives the self-assembly of the nucleoid, and regulates mitochondrial transcription, which provides a new model for mitochondria compartmentation (Long* et al.* 2021).

Herein, we summarized the compartmentation of nuclei and mitochondria in architecture assembling and discussed the phase separation from nuclei to mitochondrial nucleoids.

## MAIN

### Phase separation drives the chromatin condensation in nuclear architecture assembly

Nuclei are membrane organelles, which are responsible for chromatin-DNA storage, replication and transcription, *etc*. The chromatin-DNA is compacted by histones and condensates gradually from nucleosomes, 10 nm chromatin fiber, 30 nm chromatin fiber, euchromatin and heterochromatin to chromosomes and stored in the nuclei (Misteli 2020; Song* et al.* 2014). The chromatin-containing enhancers, especially super-enhancers could be gathered together with activators in a phase separation manner, which promotes transcription activity (Sabari* et al.* 2018). What’s more, the chromatin fiber could be locked by CTCF loops and clustered into droplets similarly driven by phase separation (Hansen* et al.* 2019). Many nuclear speckles acting as small reaction centers are generated by chromatin fiber, RNAs and so on, which are also reported to be driven by phase separation (Bi* et al.* 2019; Guo* et al.* 2019). The m6A-modification on RNA is reported to be critical for generating transcription activation speckles by phase separation (Cheng* et al.* 2021; Lee* et al.* 2021). The chromatin fiber could be further condensed by linker histone H1 and HP1a which also drives the special heterochromatin foci formation in a phase separation manner (Larson* et al.* 2017; Strom* et al.* 2017; Wang* et al.* 2020). The phase separation induces the aggregation of nucleosomes or chromatin fibers. What’s more, the condensation by phase separation in heterochromatin prevents the invasion of transcription factors, maintaining the inactivation status (Larson and Narlikar 2018; Sabari* et al.* 2020). In contrast, the euchromatin is enriched with transcription activation factors, RNA polymerase, RNAs and so on, but not H1 and HP1a, promoting gene activation and mRNA transcription.

The phase separation also provides a mechanical force for the architecture assembly and maintenance in cell nuclei. The chromatin is reported to be a gel-like structure, rather than liquid as the previous report (Strickfaden* et al.* 2020). The gel-like structure is also a feature of phase separation, which shows properties between solids and liquids. The gel-like structure of chromatin should also provide important mechanical support for the nucleus. The dynamic of the phase separation in chromatin may also regulate the mechanics of the nuclei, resulting in cell migration and invasion (Nava* et al.* 2020).

The nucleolus is the largest sub-organelle in the nuclei, which could be easily visualized under a normal microscope even in a bright field. It is a special reaction center without membrane segregation, which is responsible for rRNA transcription and ribosome assembly. It is reported to be a layered structure, starting from an inner core from rRNA transcription and proceeding toward the periphery (Brangwynne* et al.* 2011; Lafontaine* et al.* 2021). To keep the reaction smoothly and steadily, a multi-layered droplet-like liquid-liquid phase separation was found to drive the nucleolus formation (Feric* et al.* 2016; Lafontaine* et al.* 2021).

### Phase separation drives the assembly of the mitochondrial nucleoid

**Figure 1:**
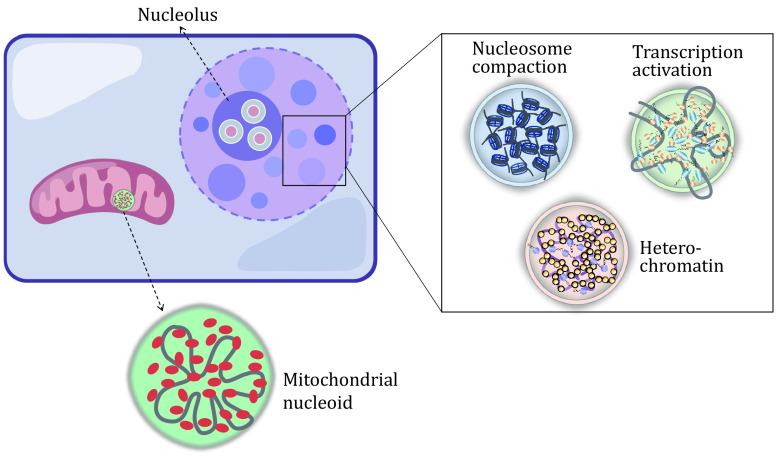
Phase separation in chromatin assembly of the nuclei and the assembly of mitochondrial nucleoid. The schematic diagram represents the phase separation in chromatin condensation and nucleus architecture. The phase separation of TFAM (red) and DNA (gray) drives the nucleoid assembly in mitochondria

Unlike nuclei, the nucleoid is a membraneless organelle in mitochondria, lacking membranes to segregate it from the mitochondrial matrix. Mitochondria are thought to be derived from ancient bacteria (Roger* et al.* 2017), which possess a simple circle DNA, compacted by TFAM proteins into a nucleoid structure, similar to the nucleoid in bacteria (Kukat* et al.* 2015). Although the nucleoid is much more relaxed than chromatins, it shares many commons with nucleosomes. The high abundance of TFAM coated mtDNA in a ratio of 1:1000 to 1:2000 as reported (Ngo* et al.* 2014; Shen and Bogenhagen 2001; Takamatsu* et al.* 2002). The core of nucleoid constructed by TFAM-mtDNA, which could also be assembled easily *in vitro* in a droplet-like manner (Farge* et al.* 2014; Feric* et al.* 2021; Long* et al.* 2021). Our data suggest that the nucleoid is self-assembled by phase separation (Long* et al.* 2021), and the flexible linker domain between two HMG domains of TFAM contributes to the phase separation of TFAM *in vitro*. The interaction between TFAM and mtDNA provides another force as the multi-covalent effect in driving the droplet-like structure of nucleoid. The phase separation promotes the segregation of nucleoids from the mitochondrial matrix, but keeps the interaction between the two of them, which should be a critical feature for mitochondria (Fig. 1).

### Phase separation regulates transcription in nuclei

**Figure 2:**
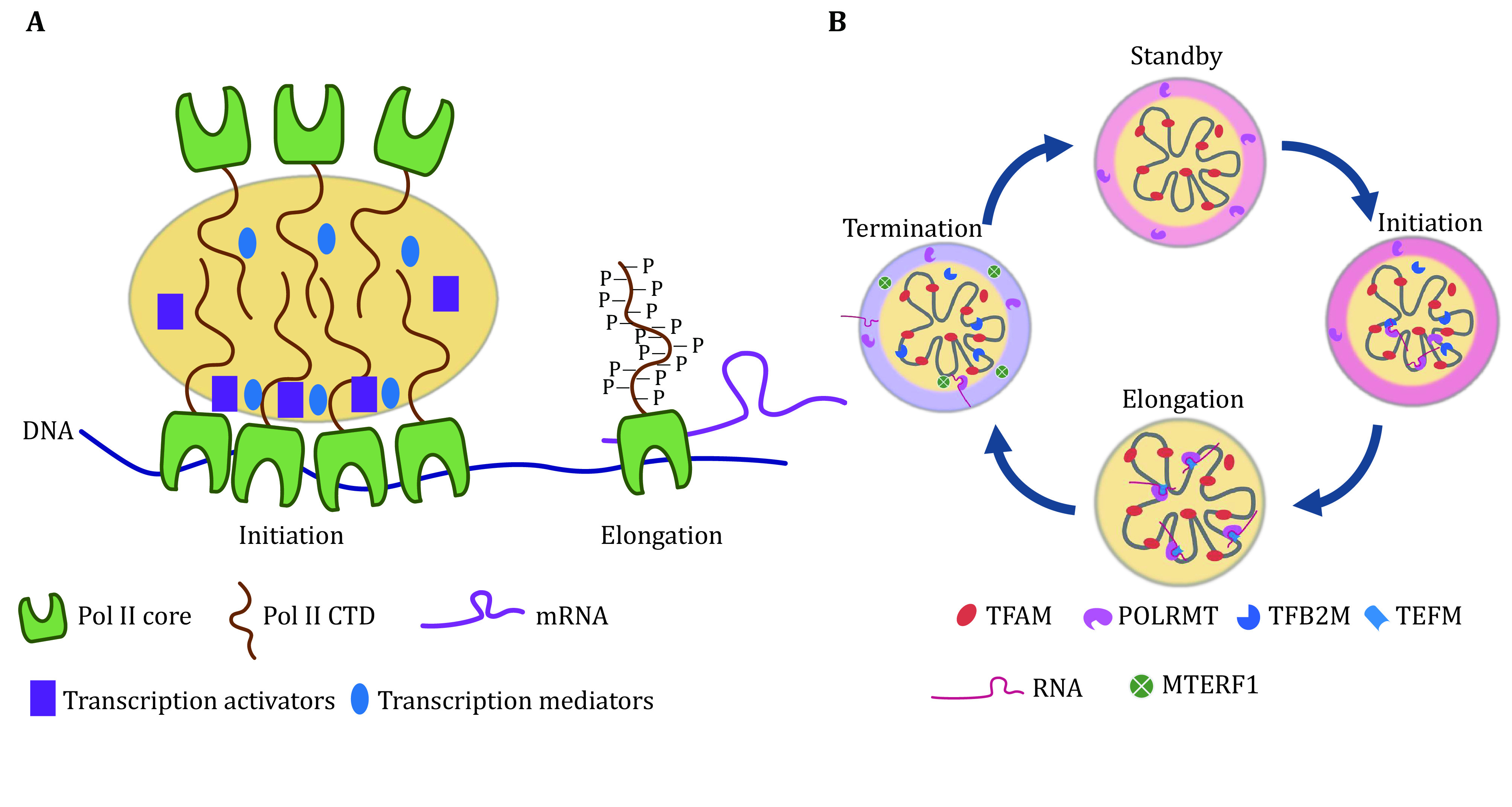
Phase separation drives transcription in nuclei and mitochondria. **A** The CTD tail (yellow) of Pol Ⅱ drives the phase separation, which recruits transcription activators (purple) and mediators (blue) in transcription initiation. The phosphorylation of CTD breaks the phase separation of Pol Ⅱ and promotes transcription elongation. **B** Phase separation in regulating transcription in mitochondria. The schematic diagram represents the phase separation in regulating mitochondrial transcription

Nuclear transcription should be carried out by multi-component transcription machinery, including hundreds of proteins (Guo* et al.* 2019; Steinbach* et al.* 2019). RNA Polymerase II (Pol II) plays a central role through its polymerase activity in mRNA generation, while the phase separation by the CTD domain also contributes to the recruitment of many other components for smooth reaction (Boehning* et al.* 2018; Guo* et al.* 2019; Lu* et al.* 2018). This CTD domain contains a conserved repeat of Y_1_S_2_P_3_T_4_S_5_P_6_S_7_, such as 52 repeats in humans, which served as an IDR for phase separation (Boehning* et al.* 2018). Other subunits necessary for transcription could be further recruited by the co-phase separation manner. The phosphorylation of CTD by CDK7 could switch the transcription from initiation to elongation by regulating the phase separation of Pol II (Boehning* et al.* 2018). The mediators were also reported to act in a similar manner of phase separation and recruit transcription activators to promote transcription or recruit repressors to inhibit transcription activity (Cho* et al.* 2018). The cis-elements, such as enhancers are also reported to be recruited by phase separation, recruiting activators and forming enhancer granules (Sabari* et al.* 2018). Moreover, transcribed RNAs could also regulate the feedback of transcription by phase separation, whereby RNAs initially stimulate condensates of transcription but then ultimately arrest the process by promoting the dissolution of these condensates (Henninger* et al.* 2020). The cleavage of pre-mRNA could also be observed as a droplet-like structure, which may be regulated by phase separation, too (Guo* et al.* 2019) (Fig. 2A).

### Phase separation in the modulation of mitochondria transcription

Mitochondria have a series of special machinery in DNA replication and transcription, different from nuclear DNA replication and transcription (Hillen* et al.* 2018). Although mitochondria originated from an ancestral prokaryotic organism, they have developed a unique transcription network to satisfy complicated demands. Mitochondrial transcription is carried out by a mitochondrial RNA polymerase (mtRNAP or POLRMT) that is more similar to single-subunit RNA polymerases (RNAPs) in bacteriophages (Masters* et al.* 1987), but is not related to multi-subunit RNAPs, such as bacterial RNAP or eukaryotic RNA polymerase II (Pol II), both exhibiting phase behavior (Boehning* et al.* 2018; Ladouceur* et al.* 2020; Lu* et al.* 2018). The bacteriophage T7 RNA polymerase, the close relative of the mitochondrial RNA polymerase POLRMT, could work independently of other factors in transcription (Durniak* et al.* 2008). However, mitochondrial transcription is a multi-component transcription and requires the assistance of additional protein factors for each step of the transcription cycle (Gustafsson *et al*. 2016; Hillen *et al*. 2018).

For the transcription initiation, mitochondrial RNA polymerase POLRMT is not able to bind to the promoter by itself (Hillen* et al.* 2017). It relies on the help of TFAM and TFB2M in promoter melting. In the nucleus, the recruitment of multi-components relies on the phase separation of a CTD domain of Pol II with 26–52 repeats (Boehning* et al.* 2018; Lu* et al.* 2018). However, POLRMT lacks such a tail, which is also not found in T7 RNA polymerase in bacteria (Long* et al.* 2021). A similar pattern of phase separation could be found on the core complex of nucleoid as TFAM-DNA (Long* et al.* 2021). Mitochondria have developed a unique way of regulating nucleoid orchestration and transcription (Fig. 2B).

The phase separation of TFAM-DNA could recruit both TFB2M and POLRMT in a co-phase separation manner, and promote efficient transcription initiation. It is a special multi-phase separation, in which TFAM-DNA presents in the core structure and POLRMT coats the core nucleoid in a shield structure. Meanwhile, TFB2M shows more flexibility and could resident both in the core and shield. This kind of special multi-phase separation could concentrate POLRMT, but also prevent the touch of POLRMT to the other part of mtDNA, which may keep the transcription in standby or low activity status. What’s more, the co-phase separation of TFB2M could promote promoter melting by opening the double-strand DNA and promoting the touch of POLRMT to mtDNA promoters, resulting in breaking the multi-phase of POLRMT and promoting transcription. The phase separation also concentrates the substrate for transcription. NTPs could be recruited by the phase separation of TFAM-DNA, which keeps the transcription efficient.

The elongation factor TEFM plays critical a role in the transformation of transcription initiation to elongation, which could also be recruited by co-phase separation with TFAM-DNA. It further remodels the multi-phase separation of POLRMT and promotes the transcription machinery to go further alongside the mtDNA.

The transcription termination in mitochondrial is still not fully determined (Terzioglu* et al.* 2013; Yakubovskaya* et al.* 2010). The terminal factors as MTERF1 are reported to bind the leu-tRNA site and terminate the transcription from the HSP promoter (Yakubovskaya* et al.* 2010). The MTERF1 could also be recruited by TFAM-DNA in a multi-phase separation manner (Long* et al.* 2021). MTERF1 circling the core complex of the nucleoid is restricted in the outer layer without the touch of most of the mtDNA-TFAM core. This kind of phase separation may also restrict the activity of MTERF1 as non-engagement with the transcription complex. Moreover, MTERF1 could co-phase separation with POLRMT in the outer layer, which may further prevent the activity of free POLRMT in the outer layer.

Similar to the transcription center in nuclei, there are also some special RNA granules in the mitochondria. Mitochondrial RNA granules are clusters of RNA transcripts and ribosome assembling (Pearce* et al.* 2017). The granules known as MRGs, are enrichment with nascent RNAs and RNA-binding proteins (Jourdain* et al.* 2013). It is reported to be an RNA processing center, in which newly synthesized RNAs are cleaved and modified. Many other proteins are also identified in the RNA granules, including GRSF1, FASTKD protein family and so on (Jourdain* et al.* 2013), which may also be driven by phase separation. Moreover, the mitochondrial ribosome assembling process is also a mystery, which may also be happened in a center driven by multi-phase separation, similar to the nucleoids.

## SUMMARY AND PERSPECTIVE

The compartmentation by phase separation provides new insight into the architecture and transcription modulation both in nuclei and the mitochondrial nucleoids. Phase separation drives chromatin condensation, nucleolus assembly and nucleus architecture construction. It also drives the mitochondria nucleoid assembly and transcription modulation.

However, there are still many questions that need to be solved. The question of how phase separation regulates chromatin dynamics in cell division and what has happened about the phase separation in control of heterochromatin building and rebuilding process in development is still uncovered. For phase separation in mitochondria, how does the transcription transform to translation in the phase separation model? Does mtDNA replication share a similar phase separation with transcription? There is still a lot of work to do as a new model and theory, and a lot of questions should be answered.

## Conflict of interest

Qi Long, Yanshuang Zhou, Jingyi Guo, Hao Wu and Xingguo Liu declare that they have no conflict of interest.
